# Prognosis of Nonagenarian ICU Patients A Bayesian analysis of prospective European studies

**DOI:** 10.1186/s13613-025-01496-2

**Published:** 2025-06-23

**Authors:** Daniel Dankl, Raphael Romano Bruno, Michael Beil, Hans Flaatten, Malte Kelm, Sviri Sigal, Wojciech Szczeklik, Muhammed Elhadi, Michael Joannidis, Andreas Koköfer, Barbara Schreiber, Franz Singhartinger, Sandra Oeyen, Brian Marsh, Rui Moreno, Susannah Leaver, Dylan W De Lange, Bertrand Guidet, Ariane Boumendil, Christian Jung, Bernhard Wernly

**Affiliations:** 1 Clinic of Anaesthesiology, Perioperative Medicine and Intensive Care Medicine, Paracelsus Medical University of Salzburg, 5020 Salzburg, Austria; 2 Medical Faculty, Department of Cardiology, Pulmonology and Vascular Medicine, Heinrich-Heine-University Duesseldorf, 40225 Düsseldorf, Germany; 3 Department of Medical Intensive Care, Hadassah Medical Center and Faculty of Medicine, Hadassah Medical Center, 91120 Jerusalem, Israel; 4 Department of Clinical Medicine, University of Bergen, Department of Anaestesia and Intensive Care, University of Bergen, 5021 Bergen, Norway; 5 Cardiovascular Research Institute Düsseldorf (CARID), Medical Faculty, Heinrich-Heine University, Duesseldorf, Germany; 6 Center for Intensive Care and Perioperative Medicine, Jagiellonian University Medical College, 31-008 Krakow, Poland; 7 Faculty of Medicine, University of Tripoli, R6XF+46G, Tripoli, Libya; 8 Division of Intensive Care and Emergency Medicine, Department of Internal Medicine, Medical University Innsbruck, 6020 Innsbruck, Austria; 9 Department of General, Visceral and Thoracic Surgery, Paracelsus Medical University of Salzburg, Salzburg, Austria; 10 Department of Intensive Care 1K12IC, Ghent University Hospital, 9000 Ghent, Belgium; 11 Mater Misericordiae University Hospital, Dublin, D07 R2WY Ireland; 12 Faculdade de Ciências Médicas de Lisboa, Nova Medical School, Centro Hospitalar de Lisboa Central, Lisbon, Portugal; 13 Faculdade de Ciências da Saúde, Universidade da Beira Interior, Covilhã, Portugal; 14 General Intensive Care, St. George’s University Hospital NHS Foundation Trust, London, SW17 0QT UK; 15 Department of Intensive Care Medicine, University Medical Center, University Utrecht, 3584 CX Utrecht, Utrecht, The Netherlands; 16 Inserm, Service de Réanimation, Sorbonne Université, Hôpital Saint-Antoine, Institut Pierre-Louis d’épidémiologie Et de Santé Publique, AP-HP, 184, Rue du Faubourg-Saint-Antoine, 75012 Paris, France; 17 AP-HP, Hôpital Saint-Antoine, Service de Réanimation, 75012 Paris, France; 18 Medical Faculty, Department of Cardiology, Pulmonology and Vascular Medicine, University Duesseldorf, Moorenstraße 5, 40225 Duesseldorf, Germany; 19 Institute of General Practice, Family Medicine and Preventive Medicine, Paracelsus Medical University of Salzburg, 5020 Salzburg, Austria; 20 Department of Internal Medicine 1, Paracelsus Medical University, Salzburg, Austria

## Abstract

**Background:**

As the population ages, the number of very elderly patients (≥ 90 years, nonagenarians) admitted to intensive care units (ICUs) is increasing. This trend raises concerns about the appropriateness of ICU care for this age group, especially due to the uncertainty surrounding their prognosis. Some studies suggest that elderly ICU patients have outcomes similar to slightly younger patients, but skepticism remains due to clinical judgment, cultural attitudes, and resource allocation concerns.

**Methods:**

We reassessed the 30-day mortality risk of nonagenarians admitted to ICUs using data from the VIP1, VIP2, and COVIP registries. Bayesian statistical methods, including Markov Chain Monte Carlo (MCMC) simulations, were used to estimate the relative risk (RR) of mortality for nonagenarians compared to octogenarians (80–89 years). Various prior assumptions (non-informative, pessimistic, and skeptical) were incorporated. The analysis adjusted for key variables such as SOFA score, frailty, and treatment limitations.

**Results:**

A total of 8,408 patients were included, consisting of 807 nonagenarians and 7,601 octogenarians. The 30-day mortality rate was 45% for nonagenarians and 42% for octogenarians (*p* = 0.12). Bayesian analysis revealed a high probability (81.1–97.9%) that nonagenarians face a higher 30-day mortality risk. However, the probability of a clinically significantly increase in mortality (RR > 1.1) was moderate (28.9–34.7%), and the probability of a substantial increase (RR > 1.2) was very low (0.03–1.9%).

**Conclusion:**

Nonagenarians in the ICU have a slightly higher 30-day mortality risk compared to octogenarians, but the increase is unlikely to exceed clinically meaningful thresholds. Bayesian methods offer more refined mortality risk assessment, suggesting that ICU admission decisions should be based on individualized factors, not just age.

## Introduction

The demographic shift towards increasing life expectancy [1] and declining birth rates in industrialized countries is reshaping healthcare demands. One consequence of this trend is the rising number of very old patients (80–90 years, octogenarians) and even older patients above 90 years of age (nonagenarians) requiring medical care, including intensive care in the case of organ dysfunction [2]. For example, the number of intensive care unit (ICU) admissions involving nonagenarians has increased 2.1-fold over a period of ten years in one study with a simultaneous 5.8-fold increase in ICU resources for this age group [3], placing significant strain on healthcare resources, both financial and personnel-related [4, 5]. Furthermore, age and frailty appear to be important risk factors for cognitive decline and post-intensive care syndrome in ICU survivors [6]. This demographic and resource challenge has sparked ongoing debate about the justification and outcomes of admitting nonagenarians to ICUs, and this debate has become a daily routine for intensive care physicians.

The reluctance of many physicians to admit nonagenarians to an ICU often stems from assumptions about their prognosis and further quality of life. While some studies suggest that elderly and very elderly ICU patients do not inherently have worse outcomes than younger patients [7–10], skepticism persists. In this content Guidet et al. showed age to be an independent factor for the decicion to withhold or withdraw life-sustaining treatment in patients admitted to the ICU [11]. Another study by Ducos et al. reported similar findings, identifying age as an independent risk factor influencing the decision-making process to limit or withdraw life-sustaining treatment in ICU patients. Notably, age alone was not found to be a risk factor for mortality in this study [12]. This skepticism is often influenced by subjective clinical judgment, cultural attitudes toward aging, and the perceived value of resource allocation. These factors complicate the decision-making process, highlighting the need for evidence that bridges empirical data and clinical intuition.

As previously noted Bruno et al. [8] evaluated the 30-day outcomes of nonagenarians admitted to ICUs. Based on frequentist statistical methods, their findings indicated no significant increase in mortality for nonagenarians after adjusting for confounders. However, their analysis did not address the potential influence of physician skepticism or the uncertainty surrounding subjective prognosis assessments. Such skepticism, not inherently reflected in clinical data, may play a critical role in shaping ICU admission decisions for this special patient population.

Bayesian methods offer a unique opportunity to address these gaps by integrating empirical data with prior beliefs and clinical intuition [13]. This Bayesian approach integrates prior assumptions—so-called'priors'—which may include physician attitudes or historical data, with current observed data, providing insights into the interplay between clinical judgment and empirical evidence [14]. This is particularly relevant in the context of nonagenarian ICU patients, where decision-making often involves balancing empirical evidence with subjective considerations.

This study aims to re-evaluate the findings of Bruno et al. [8] from the VIP1 and VIP2 cohort augmented by the COVIP cohort using Bayesian analytical methods. By incorporating Bayesian methods, we aim to provide a more comprehensive understanding of the outcomes for nonagenarian ICU patients, accounting for both objective data and the uncertainty inherent in clinical decision-making. The results of this analysis may inform ICU admission policies, optimize resource allocation, and enhance evidence-based approaches to clinical decision-making for very elderly patients.

## Methods

### Study subjects

This analysis included patients from three large registries: VIP-1, VIP-2, and COVIP (6,7,12). These registries prospectively collected data on critically ill elderly patients during distinct time periods. Specifically, 3830 patients were enrolled in VIP-1 between October 2016 and May 2017, 3770 in VIP-2 from May 2018 to May 2019, and 2754 in COVIP between March 2020 and May 2021 [8, 15–17]. The COVIP cohort included critically ill COVID-19 patients aged ≥ 70 years from 138 ICUs across 28 countries. Previous studies have analyzed the VIP1, VIP2, and COVIP registries in relation to various outcomes in intensive care. However, the primary contribution of our study is the application of a novel statistical method, Bayesian analysis.

For this study, we analyzed 8408 unique acute ICU admissions with complete data on 30-day mortality and age at admission. Data completeness was high, with missing data accounting for less than 1%. Patients from VIP-1 admitted to the ICU after elective surgery (n = 935) were excluded from the analysis. Patient characteristics are shown in Table 1.

**Table 1:** Clinical characteristics and outcomes of octogenarians (≥ 80 to < 90 years) and nonagenarians (≥ 90 years) admitted to the ICU. Data are presented as median (interquartile range) for continuous variables (analysed using the Mann–Whitney U test) or percentage (absolute number) for categorical variables (analysed using the chi-square test). Statistical significance was defined as a p-value < 0.05. (CFS = Clinical Frailty Scale, Score > 4 indicates frailty)

	Octogenarian (≥ 80 to < 90)	Nonagenarian (≥ 90)	*p*-value
	N = 7601	N = 807	
Age (years)	83 (81–86)	91 (90–93)	< 0.001
SOFA score	7 (4–10)	6 (4–9)	< 0.001
Male sex	55% (4161)	43% (349)	< 0.001
Non-invasive ventilation	25% (1931)	21% (171)	0.008
Mechanical ventilation	53% (4007)	42% (337)	< 0.001
Use of vasoactive drugs	59% (4479)	52% (417)	< 0.001
Renal replacement therapy	12% (874)	4% (34)	< 0.001
ICU length of stay (median hours)	89	54	< 0,001
Trachostomy performed	7,38% (301)	3,62% (15)	0,004
Withhold therapy order	29% (2204)	36% (286)	< 0.001
Withdraw therapy order	16% (1178)	13% (107)	0.085
30-day-mortality	42% (3219)	45% (365)	0.120
Number of chronic comorbidities	4 (3–6)	4 (3–5)	0.640
CFS	4 (3–6)	5 (4–6)	< 0.001
CFS ≤ 4	59% (4407)	42% (337)	
CFS > 4	41% (3100)	58% (462)	

### Statistical analysis and software

Bayesian priors were established following a scoping review of the existing literature, which revealed a lack of high-level evidence to guide their selection. In response, we adopted a pragmatic approach informed by clinical judgment within the study group. A non-informative prior was defined with a mean of 0 and a standard deviation (SD) of 10 on the log scale, ensuring minimal constraints on the data. Additionally, we specified a pessimistic prior with a mean of ln(1.1) and an SD that included a 5% probability of no effect, reflecting the clinical assumption that a 10% increase in 30-day mortality represents a meaningful threshold. A skeptical prior was defined with a mean of 0 and an SD that included a 5% probability of relative risks exceeding 1.1, acknowledging uncertainties about the presence of a true effect. Given the clinical plausibility that 30-day mortality is unlikely to be lower in nonagenarians compared to octogenarians, we did not include an optimistic prior.

Bayesian analyses were performed using Stata’s Markov Chain Monte Carlo (MCMC) methods with 12,500 iterations, including a burn-in phase of 2500 iterations. Convergence was assessed using standard diagnostics. Relative risks (RRs) were estimated via Poisson regression with robust standard errors. We chose RRs over odds ratios due to the high event rate and the better interpretability of RRs [18]. Two models were constructed: an unadjusted model (Model-1) and a fully adjusted model (Model-2) controlling for sex, Sequential Organ Failure Assessment (SOFA) score, Clinical Frailty Scale (CFS), non-invasive ventilation (NIV), mechanical ventilation (MV), renal replacement therapy (RRT), admission diagnosis, and treatment limitation decisions. Posterior distributions were calculated for RRs, and their densities were visualized to explore the range and uncertainty of the estimated effects. We assessed convergence using the Gelman–Rubin diagnostic (potential scale reduction factor, PSRF) across four independent MCMC chains, each with 10,000 iterations. All Rc values were well below the commonly accepted threshold of 1.1 (maximum Rc = 1.0008), indicating satisfactory convergence.

To assess the clinical relevance of the findings, posterior probabilities were calculated for thresholds of relative risk > 1.0, > 1.1, and > 1.2. These thresholds reflect benchmarks for any increased risk, clinically meaningful risk, and substantial risk, respectively. Additionally, the area under the curve (AUC) was computed for each threshold, providing a concise summary of the posterior evidence supporting these outcomes.

Descriptive statistics were used to characterize the cohort. Continuous variables were reported as medians with interquartile ranges (IQRs), given the non-normal distribution of most biomarkers. Categorical variables were summarized as counts and percentages (n, %). These analyses provided context for the Bayesian models by highlighting the distribution of key covariates in the study population, including frailty and SOFA scores. All analyses were performed using Stata/BE, version 18.5 (StataCorp LLC, College Station, TX, USA). All data is available at reasonable request. ChatGPT was used for the linguistic revision of the manuscript. The tool had no influence on the content or scientific analysis of the work presented.

## Results

A total of 8408 patients were included in the study, comprising 807 nonagenarians (≥ 90 years) and 7601 octogenarians (≥ 80 to < 90 years). The median age was 91 years (IQR 90–93) for nonagenarians and 83 years (IQR 81–86) for octogenarians. The SOFA score was slightly lower in nonagenarians (median 6, IQR 4–9) compared to octogenarians (median 7, IQR 4–10, *p* < 0.001). Clinical frailty scale (CFS) grading was significantly higher in the nonagenarian group [nonagenarians 5 (IQR 4–6) vs. octogenarians 4 (IQR 3–6)] with more nonagenarians having a CFS grading greater than 4.

In terms of sex distribution, 43% of nonagenarians were male, compared to 55% of octogenarians (*p* < 0.001). Non-invasive ventilation (NIV) was used in 25% of octogenarians and 21% of nonagenarians (*p* = 0.008). Intubation and mechanical ventilation were more common among octogenarians (53% vs. 42%, *p* < 0.001), as was the use of vasopressor drugs (59% vs. 52%, *p* < 0.001). Renal replacement therapy (RRT) was initiated in 12% of octogenarians compared to 4% of nonagenarians (*p* < 0.001). The decision to withhold treatment was more frequent in nonagenarians (36% vs. 29%, *p* < 0.001), whereas withdrawal of treatment was similar between the groups (16% vs. 13%, *p* = 0.085).

**Graph 1:**
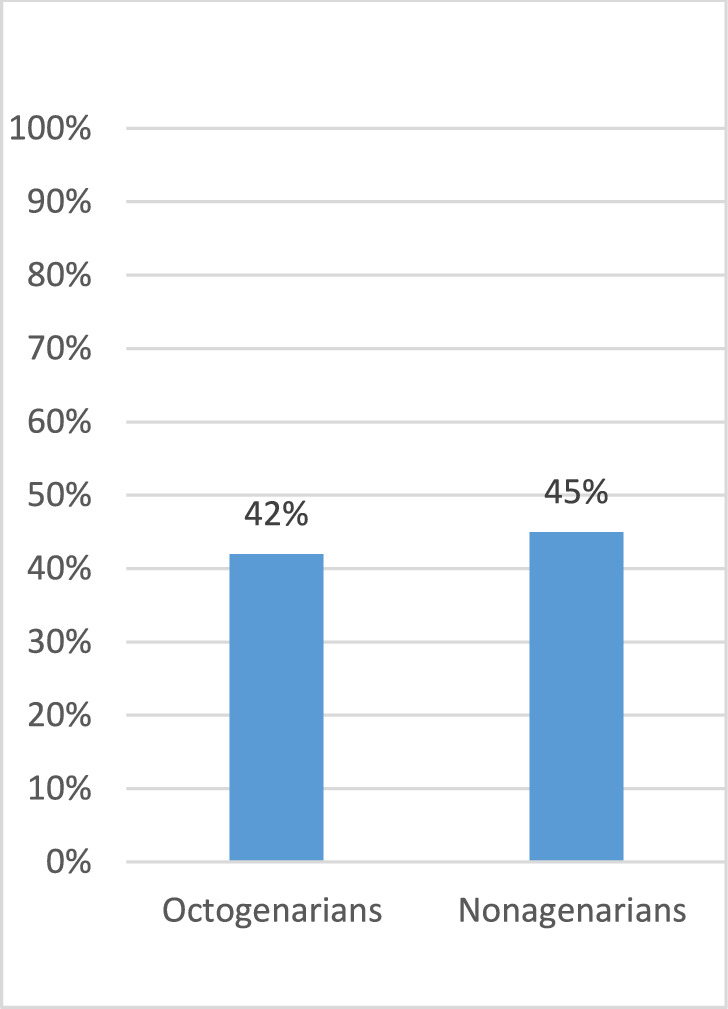
Comparison of 30-day mortality between octogenarians (80–89 years) and nonagenarians (≥ 90 years) using frequentist statistical analysis. Bars represent mortality rates

The 30-day mortality rate was 45% (365) among nonagenarians and 42% (3219) among octogenarians, resulting in an absolute difference of 3 percentage points (*p* = 0.120). Both groups had a similar burden of chronic comorbidities, with a median of 4 comorbidities (*p* = 0.640) (Table 1; Graph 1).

**Graph 2:**
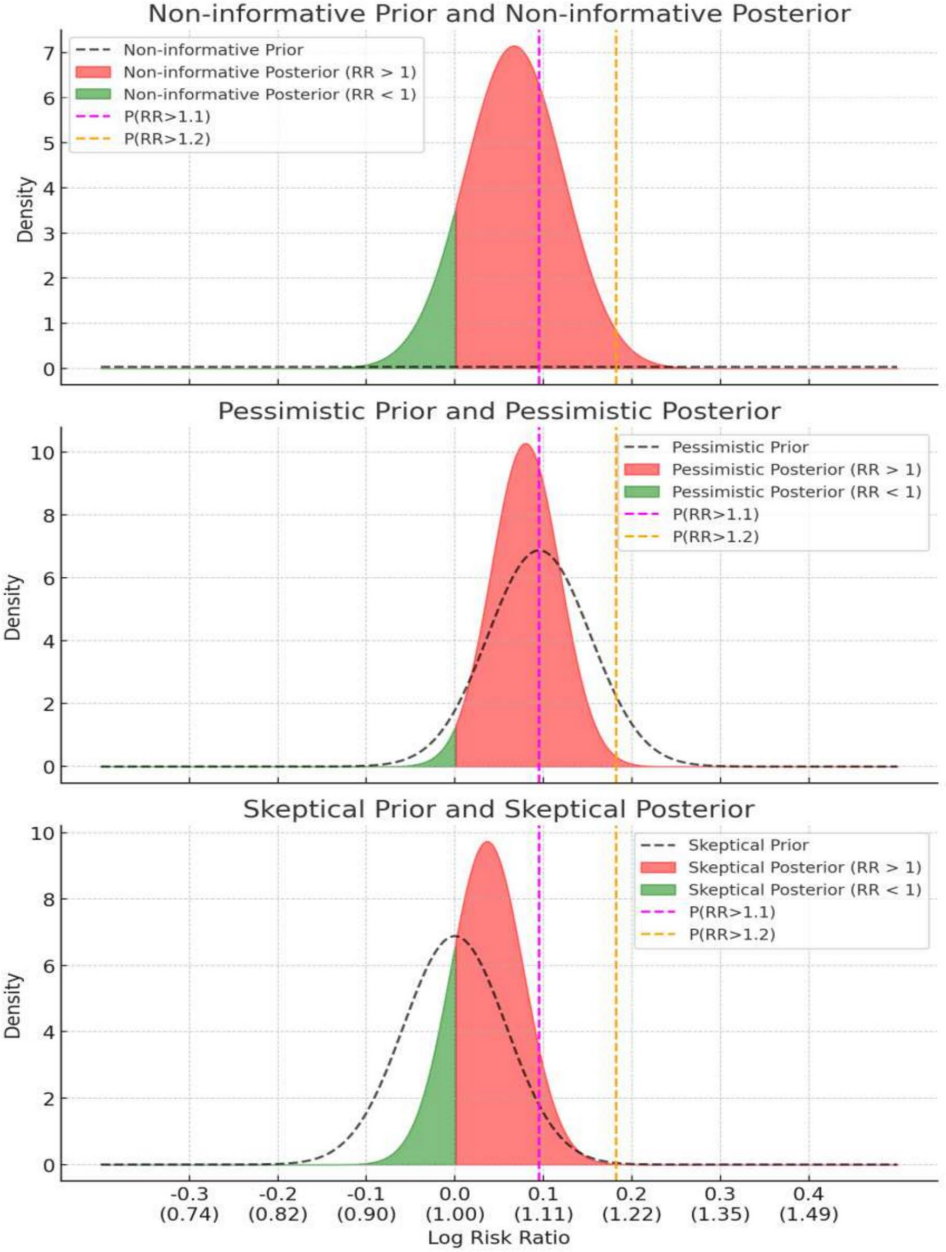
Prior and posterior distributions for Bayesian analyses of three different priors: “Non-informative Prior” (mean = 0, SD = 10), “Pessimistic Prior” (mean = 0.095, SD = 0.058), and “Skeptical Prior” (mean = 0, SD = 0.058). Posterior distributions reflect unadjusted analyses with respective medians and 95% highest posterior density intervals. Shaded areas under the posterior curves are red for RR > 1 and green for RR < 1. Vertical dashed lines at ln(1.1) (magenta) and ln(1.2) (orange) indicate thresholds for P(RR > 1.1) and P(RR > 1.2). Log Risk Ratio values and corresponding Risk Ratios are shown on the x-axis

The unadjusted Bayesian analysis (Model-1) (Graph 2) estimated the relative risk (RR) of 30-day mortality for nonagenarian ICU patients under three prior assumptions. Using the non-informative prior, the RR was estimated to have a 95% highest posterior density credible interval (HPD CrI) of 0.946 to 1.177. The probabilities of the RR exceeding 1.0, 1.10, and 1.20 were 0.882, 0.290, and 0.018, respectively. Under the pessimistic prior, the RR was estimated with a 95% HPD CrI of 0.999 to 1.163, and the probabilities of the RR exceeding 1.0, 1.10, and 1.20 were 0.980, 0.347, and 0.005. Using the skeptical prior, the RR was estimated with a 95% HPD CrI of 0.953 to 1.119, and the probabilities of the RR exceeding 1.0, 1.10, and 1.20 were 0.811, 0.070, and 0.000, respectively (Table 2).

**Table 2:** 95% Highest posterior density credible intervals (HPD KrI) and probabilities for risk ratios [P(RR)] with the non-informative, pessimistic and skeptic prior in the unadjusted and adjusted model

	(95% HPD KrI)	P(RR > 1)	P(RR > 1.10)	P(RR > 1.20)
Model-1 (unadjusted)				
Non-informative	(0.946—1.177)	0.882	0.290	0.018
Pessimistic	(0.999—1.163)	0.979	0.347	0.005
Skeptic	(0.953—1.119)	0.811	0.070	0.000
Model-2 (adjusted)				
Non-informative	(0.985—1.225)	0.953	0.499	0.061
Pessimistic	(1.013—1.184)	0.992	0.493	0.015
Skeptic	(0.967—1.136)	0.896	0.142	0.001

**Graph 3:**
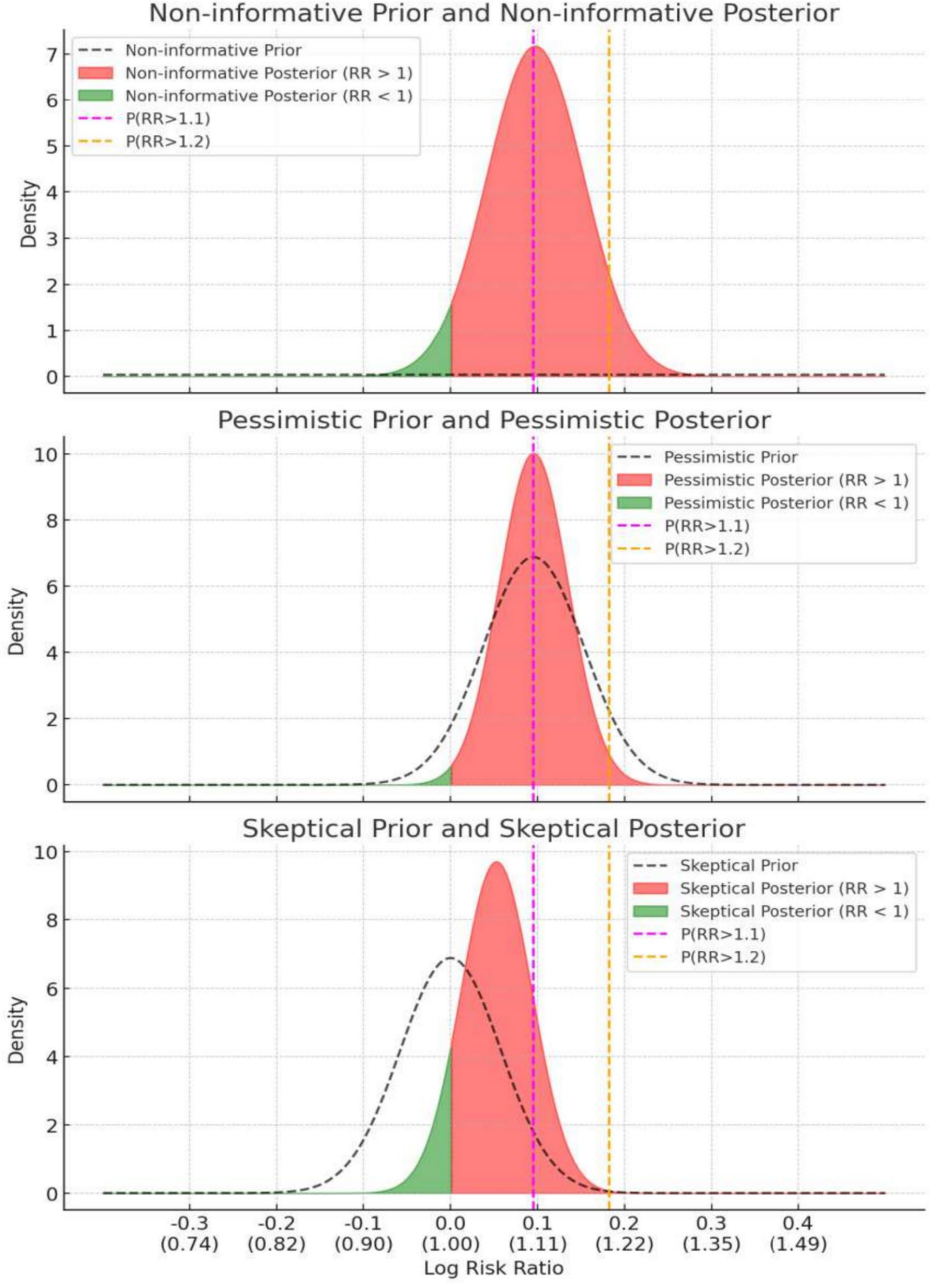
Prior and posterior distributions for Bayesian analyses of three different priors: “Non-informative Prior” (mean = 0, SD = 10), “Pessimistic Prior” (mean = 0.095, SD = 0.058), and “Skeptical Prior” (mean = 0, SD = 0.058). Posterior distributions reflect adjusted analyses with respective medians and 95% highest posterior density intervals. Shaded areas under the posterior curves are red for RR > 1 and green for RR < 1. Vertical dashed lines at ln(1.1) (magenta) and ln(1.2) (orange) indicate thresholds for P(RR > 1.1) and P(RR > 1.2). Log Risk Ratio values and corresponding Risk Ratios are shown on the x-axis

The adjusted Bayesian analysis (Model-2) (Graph 3) provided estimates for the relative risk (RR) of 30-day mortality under three prior assumptions. With the non-informative prior, the RR was estimated with a 95% highest posterior density credible interval (HPD CrI) of 0.985 to 1.225, and the probabilities of the RR exceeding 1.0, 1.10, and 1.20 were 0.953, 0.499, and 0.061, respectively. Using the pessimistic prior, the RR had a 95% HPD CrI of 1.013 to 1.184, with probabilities exceeding 1.0, 1.10, and 1.20 at 0.992, 0.493, and 0.015, respectively. Under the skeptical prior, the RR was estimated with a 95% HPD CrI of 0.967 to 1.136, and the probabilities of the RR exceeding 1.0, 1.10, and 1.20 were 0.896, 0.142, and 0.001, respectively (Table 2).

## Discussion

In this study, we used Bayesian analysis to compare the 30-day mortality rates between nonagenarians and octogenarians admitted to the ICU. Our findings suggest that while nonagenarians may have a slightly higher mortality rate compared to octogenarians, the probability of a clinically significant increase in mortality (greater than 10%) is low. Even under pessimistic assumptions, the likelihood of a mortality disadvantage greater than 20% was almost negligible. These results challenge the common assumption that ICU treatment for nonagenarians is inherently futile and suggest that age alone should not be the sole criterion for ICU admission decisions.

As the number of very old patients admitted to the ICU continues to rise prognostic assessment in this population is particularly challenging due to limited literature and the absence of validated, evidence-based decision-supporting tools. The VIP1 and VIP2, as well as the COVIP study groups, have discussed in several papers [19–21] that prognostic assessment for very elderly patients is particularly difficult due to the inadequacy of conventional prognosis scores for this patient group. Key factors such as frailty and multimorbidity are not included in traditional prognostication scores like the SOFA score. In particular, the VIP study group has demonstrated that frailty has a decisive impact on the prognosis of very elderly intensive care patients.

Moreover, aspects like long-term quality of life and the risk of post-intensive care syndrome are not addressed in conventional prognosis models. It is also unclear whether age alone is a risk factor for reduced quality of life after an intensive care unit stay. For example, Takita [22] demonstrated in a cohort of 96 Japanese intensive care patients that older age (mean 78 vs. 51 years) was not associated with an increased risk of post-intensive care syndrome. Of course such data must also be interpreted in light of the cultural and country-specific demographic situation and cannot always be applied to other patient populations.

As a result, prognostic assessment and thereby decision-making regarding ICU care or withholding/withdrawing therapy for very elderly patients often relies heavily on the clinical judgment of the treating physicians. And as previously mentioned, emotional, personal, and cultural factors can have a significant influence on treatment decision-making for very elderly patients [23].

Despite these specific challenges in prognosis assessment for very elderly patients and the limited research on this topic, especially regarding the prognosis of nonagenarians in comparison to slightly younger patients, some of the sparse available data suggest that survival outcomes for nonagenarians in the ICU are not markedly worse than those for younger individuals. For instance, Roedl et al. [7] found that nonagenarians who had a return of spontaneous circulation (ROSC) after cardiac arrest outside the ICU and were then treated in the ICU showed relatively good neurological outcomes, with 46% ICU survival and a high rate of favorable outcomes. Similarly, Garrouste-Orgeas et al. [24] analysed data from 2419 ICU patients and found no significant difference in ICU or in-hospital mortality between nonagenarians and octogenarians in a matched cohort. Interestingly, ICU and hospital stays were shorter for nonagenarians in this study. Of course, it must be considered that this does not necessarily indicate a good medium- and long-term prognosis for these patients.

Additionally, Bruno et al. [8] examined the VIP1 and VIP2 registries, which included 7900 elderly critically ill patients, and found no statistically significant difference in ICU or 30-day mortality between nonagenarians and octogenarians after adjusting for factors such as SOFA score, frailty, age, and sex.

While these studies used frequentist statistics, indicating no significant difference in mortality between nonagenarians and octogenarians, clinical practice often requires more specific probabilities to guide decision-making. For instance, a physician may find it more relevant to understand the likelihood of a particular difference in mortality rather than simply knowing there is no significant statistical difference in mortality overall. This might especially be true in the complex situation of decision-making about nonagenarians in an ICU.

So traditional frequentist methods indicate no statistically significant difference in 30-day mortality between nonagenarians and octogenarians in some studies. However, in clinical practice, binary significance testing offers limited utility. Physicians require explicit probabilities to guide decision-making.

Bayesian analysis addresses this gap by estimating the probability of a clinically meaningful difference, rather than merely testing for statistical significance. This approach enables a more nuanced interpretation, allowing intensivists to weigh risks and benefits more effectively when making critical treatment decisions. This is achieved by combining previous research and clinical experience with actual data from studies. This approach can offer more tailored, patient-specific information, particularly for complex cases such as those involving nonagenarians on the ICU. Bayesian methods also allow for direct calculation of the probability of certain hypotheses, such as an increased percentage-wise higher 30-day mortality risk for nonagenarians compared to octogenarians.

Non-informative, pessimistic and skeptical priors were defined to perform the Bayesian analysis. Due to the limited available literature, the priors were selected in such a way that a 10% increase in 30-day mortality was considered clinically significant. In the absence of data, the prior was defined with a threshold of 1.1, representing a 10% higher mortality, based on the clinical judgment of the study authors, representing extensive cumulative experience in intensive care, that such mortality difference would possibly change clinical decision making. Determining what constitutes a “least worthwhile effect” [25] is inherently challenging and may vary depending on the perspective of both the patient and the physician. Therefore, our assumption of a 10% higher mortality for nonagenarians and our estimation of the likelihood of such an effect represent a pragmatic approach to assist intensive care physicians in making decisions based on more concrete numbers in everyday clinical practice.

In the bayesian analysis, all calculations show a slightly higher mortality rate for nonagenarians compared to octogenarians. However, it is unlikely that this mortality disadvantage exceeds the established clinically significant threshold of 10%, depending on the prior. Even under the assumption of the pessimistic prior, the probability of a higher mortality of at least 10% for nonagenarians compared to octogenarians is 43.7% in the unadjusted model and 49.2% in the adjusted model. The probability of a mortality disadvantage of at least 20% is virtually zero in both models (unadjusted and adjusted).

Since frailty has been shown to be one of the crucial determinants of mortality risk in elderly and very elderly intensive care patients [14, 26–29] our model was adjusted for frailty. Treatment limitations as “withhold therapy” and “withdraw therapy” were also both included in the adjusted model as treatment limitation decisions are also often very subjective and based on the personal experience and cultural background of the treating physicians [30]. It is, of course, not entirely clear to what extent the factors of frailty and treatment limitations may influence each other in a self-fulfilling prophecy manner. Investigating this would likely require a study design in which treatment limitations are applied independently of clinical judgement, a scenario that would be ethically at the very last challenging to implement. Therefore, we tried at least to reduce the influence of these factors by adjusting for them.

A major limitation of this study is that the cohorts include patients who were already admitted to the intensive care unit, meaning there was a pre-selection process either in the general ward or the emergency department leading to ICU admission. This means that only a few nonagenarians might have been admitted to an ICU. This phenomenon has been shown by Sprung et al. [31] who showed a very high ICU refusal rate of patients over 85 years of age. This pre-selection of very elderly patients might be highly subjective, as determined by the treating physicians, which was demonstrated in a study by Boumendil et al. [32] who showed that age and previous functional status were the major determinants of ICU refusal. This limitation must be taken into account when interpreting and applying the results of our study, particularly in cases where the decision regarding ICU admission has not yet been made.

For the same reason, elective surgical patients were excluded from our analysis as Jung et al. [33] showed a favourable outcome in very old elective surgery patients when compared to emergency surgery patients of the same age group.

A further limitation on the other hand might be the fact that a pre-selection bias among nonagenarians cannot be excluded. Some clinicians may argue that nonagenarians must be relatively healthy, as otherwise, they would not have lived to such an advanced age. There is some literature supporting this consideration [34]. Such perception could potentially mitigate the aforementioned selection bias. However, frailty was slightly higher in nonagenarians in the analysed cohort so that this argument might be challenged. On the other hand, although the mortality rate was relatively high in both the nonagenarian (45%) and octogenarian (43%) groups, indicating that there may not have been a strong preselection of the fittest patients, the SOFA score was slightly lower in the nonagenarians (6 vs. 7, *p* < 0.001). Additionally, in nonagenarians there were fewer vasopressors used (52% vs. 59%, *p* < 0.001) and they were less frequently invasively ventilated (42% vs. 53%, *p* < 0.001). However, this might also be attributed to the higher rate of withheld therapy orders in the nonagenarians. To account for these potential confounders, we performed the adjusted analysis, showing the aforementioned results.

Although the adjusted models incorporated important covariates such as frailty and treatment limitations, the resulting credible intervals largely overlapped with those of the unadjusted models. This indicates that the adjustment did not substantially change the conclusions regarding mortality risk differences between octogenarians and nonagenarians. The probability estimates for a clinically meaningful increase in mortality (e.g., RR > 1.10) remained similar across both models, regardless of the chosen prior. This suggests that while frailty and treatment limitations are relevant clinical factors, their statistical impact on the observed mortality difference in our cohort appears limited.

Another limitation of our study is the lack of detailed information regarding the specific diagnoses or underlying pathologies that led to ICU admission. While we adjusted for disease severity using the SOFA score, which captures the extent of organ dysfunction, we acknowledge that different admission diagnoses may influence both outcomes and treatment decisions. Future research should aim to incorporate more granular diagnostic data to better contextualize the relationship between age, disease severity, and prognosis in critically ill elderly patients.

We also acknowledge that our study focuses on 30-day mortality as the primary outcome, without capturing longer-term outcomes such as functional decline or post-intensive care disability, which are particularly relevant in elderly patients. While we adjusted for key prognostic factors including SOFA score, frailty, and treatment interventions, other relevant predictors of poor prognosis may not have been captured. A more comprehensive assessment of functional outcomes and long-term disability would strengthen the clinical utility of prognostic models and should be a focus of future studies in this population.

Aside from these limitations our findings provide critical insights for intensivists facing difficult decisions about ICU admissions for very elderly patients. Bayesian analysis reinforces that age alone should not be the determining factor in ICU admission decisions. Rather than relying solely on traditional significance testing, incorporating probabilistic models into clinical practice may enable more personalized and evidence based decision-making. Furthermore, our findings challenge the perception, sometimes encountered in clinical practice, that ICU treatment in nonagenarians is of limited benefit. Moreover, it underscores the need for individualized assessments rather than rigid age-based admission criteria. Future research should focus on refining prognostic models that integrate frailty, treatment limitations, and long-term quality-of-life outcomes to provide clearer guidance for intensivists managing very elderly patients.

## Conclusion

While nonagenarians admitted to ICUs may have a slightly higher 30-day mortality risk compared to octogenarians, our Bayesian analysis suggests that this difference is unlikely to exceed a clinically meaningful threshold. Our findings challenge the assumption that ICU admission for nonagenarians is inherently futile and highlight the need for individualized, evidence-based decision-making. By integrating Bayesian probabilities into prognostic assessments, clinicians can make more informed treatment decisions, reducing reliance on subjective biases and ensuring a more nuanced evaluation of ICU eligibility in very elderly patients. This leads to the conclusion that the decision to admit nonagenarians to the ICU should not be based solely on age since age alone does not seem to be a relevant prognostic factor in a clinically meaningful manner. Furthermore, it appears valuable to develop combined tools utilizing the available data that would enable clinicians to assess the prognosis of very elderly patients, both in the emergency department and in the intensive care unit. A combination of organ dysfunction scores, such as the SOFA score, frailty assessments, and individual subjective clinical judgment, would be suitable for this purpose.

## Data Availability

Data and material are available on reasonable request.
